# Volume of Treatment of Temporomandibular Disorders in Adults—A Nationwide Register‐Based Observation of Private Oral Healthcare in Finland in 2006–2024

**DOI:** 10.1111/joor.70272

**Published:** 2026-07-14

**Authors:** Miira M. Vehkalahti, Kaija Hiltunen, Maria Valaste

**Affiliations:** ^1^ Department of Oral and Maxillofacial Diseases, Faculty of Medicine University of Helsinki Helsinki Finland; ^2^ Centre for Social Data Science, Faculty of Social Sciences University of Helsinki Helsinki Finland

**Keywords:** adults, occlusal splint, oral healthcare, private dentistry, register‐based study, temporomandibular disorders

## Abstract

**Background:**

Temporomandibular disorders (TMD) present diverse signs and symptoms and often require clinical evaluation.

**Objective:**

To investigate variation in the volume of TMD treatment among adult patients in Finnish private oral healthcare between 2006 and 2024.

**Methods:**

This retrospective register‐based study used annual aggregate data from the Social Insurance Institution of Finland on patients aged 20 years or older. Per year, an average of 56 500 patients received TMD‐related procedures, which were primarily coded by visit duration; only splint therapy was coded as a single, specific procedure. TMD treatment volume was described as the number and proportion of TMD patients and procedures, overall in each year from 2006 to 2024 and in detail, by 5‐year age groups in selected years (2012, 2014, 2017, 2020, 2022 and 2024). Linear regression assessed associations between treatment year, age group and treatment volume.

**Results:**

From 2006 to 2024, TMD patients constituted 4.1%–6.7% of all patients, peaking in 2015–2019 and in age groups 25–55 years. TMD procedures accounted for 1.2% of all clinical procedures in 2006, 2.4% in 2020 and 2.0% in 2024. Short‐ and medium‐duration TMD procedures predominated; splint therapy was more common in younger patients (in 2024, 28%–32% of TMD patients aged 20–39 years and 25%–27% aged 40–54 years). Linear regression showed strong, negative associations between age group and TMD treatment volume (*p* < 0.001).

**Conclusion:**

TMD therapy is more common among younger adults. Register‐based data indicate increasing use of splint therapy, particularly among younger patients. Recent declines in overall volume after peak years may reflect changes in clinical decision‐making and promote a more nuanced understanding of treatment risks.

## Graphical abstract

This study established a long‐term picture of TMD treatment in private oral healthcare; the data covered approximately 56 500 TMD patients per year from 2006 to 2024. TMD patients constituted 4.1%–6.7% of all patients; 13.9% of TMD patients received splint therapy, most commonly among younger adults. No noticeable changes appeared during the COVID‐19 period.

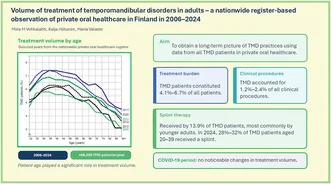

## 1 Introduction

Temporomandibular disorders (TMD) comprise a multifaceted set of symptoms with variable presence and severity. TMD signs and symptoms are common in both clinically examined patient populations and in general population studies. According to Carlsson [1], TMD signs affect about 30%–60% of the population, but only a fraction (5%–12%) require treatment. In northeastern Germany, 49.9% of 20–79‐year‐old residents in a rural region had one or more clinical signs of TMD, while only 2.7% were subjectively aware of joint pain [2]. In Finland, 38% of the adults clinically examined in a nationwide population study in 2000 had at least one sign of TMD [3], and 35% did so in 2011 [4]. In Sweden, a public‐sector survey of adult patients in Västerbotten County found that 4%–5% of men and 8%–12% of women reported experiencing TMD symptoms or pain at least once a week [5], while in Stockholm, 4.6% of the patients in 2015–2019 responded affirmatively to at least 1 of the 3 questions on signs and symptoms of TMD [6]. In Italy, a survey of adults found that 30.7% reported TMD clicking and 16.3% reported TMD pain, most common in the 45–60‐year age group [7]. A recent worldwide meta‐analysis reports a global prevalence of TMD of 26% in North America, 29% in Europe, 33% in Asia and 47% in South America [8], while a very recent systematic review reported corresponding estimates of 19.4%, 33.8%, 27.9% and 27.3%, respectively [9].

Because a patient's perceived need for care varies from day to day, symptoms and signs may be missed during routine oral health examinations. Evidence on TMD treatment needs often relies on clinical examinations or questionnaires; however, there is limited information on routine treatment provision. A meta‐analysis estimated that treatment need in the general adult population is around 15.6%–16.2% and varies by definition, criteria and age [10]. Still, little is known about TMD treatments in everyday practice. A study from Västerbotten, Sweden, reported that 6.1% of patients identified as TMD cases received occlusal appliance therapy [11]. In the Stockholm study, 18.3% of those who answered affirmatively received TMD treatment [6]. Based on a Finnish report of total treatment counts [12], it can be calculated that among adults in the private oral healthcare services, TMD treatments constituted 1.3% of all procedures and 6.4 TMD procedures per 100 patients. The corresponding figures for the public sector were 0.6% and 3.2 per 100 patients.

To obtain a long‐term picture of TMD practices, this study assessed the number of patients and procedures recorded annually in official statistics in subsidised private oral healthcare. Specifically, it examined variation in the volume of TMD treatment received by adult patients in private oral healthcare in Finland between 2006 and 2024. The null hypothesis was that the number of TMD patients would change annually in parallel with changes in the total number of patients.

## 2 Methods

The target population comprised all adult patients, aged ≥ 20 years, using private oral healthcare services in 2006–2024. The Social Insurance Institution of Finland (SII) maintains patient‐based registry information and aggregates it by calendar year. For each year, the data cover services provided from 1 January to 31 December [13]. As the data were aggregated, no patient identities were available; therefore, no ethics approval was required. This retrospective register‐based observational study used annual pooled SII data on adults receiving TMD treatment between 2006 and 2024. The register‐based data were pre‐aggregated into 5‐year age groups and included all patients aged 20 years or older. No sampling was performed because the data encompassed counts of all adult patients and treatment procedures by year.

A patient was defined as a user of private oral healthcare if they had received at least one treatment procedure during the year in question, and as a TMD patient if they received one or more TMD procedures. The data included key information for each year, from 2006 to 2024, for a total of 19 years. In addition, detailed information about TMD treatments was collected over the selected years to capture pre‐ and post‐COVID‐19 pandemic situations and potential changes during the pandemic in 2020–2022. The selected years were 2012, 2014, 2017, 2020, 2022 and 2024. Data were organised by 5‐year age groups from 20–24 to 85–89, with all 90+ combined.

The Finnish Institute for Health and Welfare (THL) defines the codes required for recording TMD procedures. Complete check‐ups include recording TMD status, which, if a TMD diagnosis is made, may lead to initiating TMD treatment. TMD codes are used only for recording TMD treatments. The definitions of procedure codes are published and regularly updated [14]. All TMD procedures performed during a single visit that are not separately classified elsewhere are recorded by visit duration, while only splint therapy is coded as a single, specific procedure. Four original TMD codes were duration‐based (≤ 10, ≤ 20, ≤ 30, > 30 min), and one comprehensive code covered splint therapy (fabrication, fitting, adjustments, instructions, follow‐up). In 2021, new codes for increasing occlusal vertical dimension were added for procedures deemed necessary in certain TMD cases. In the analysis, the codes were grouped as short (≤ 10 min), medium (≤ 20 min), long (> 20 min), and splint therapy or increasing occlusal vertical dimension procedure.

### 2.1 Statistical Analysis

The observation unit was the patient group per year of treatment; subgroups were 5‐year age groups. Volume of TMD treatment was described as the number of TMD patients among all patients, the proportion of TMD procedures among all procedures, and the proportion of splint procedures among TMD procedures. Percentages of TMD patients and mean numbers of procedures were calculated by treatment year and by age group. Changes from 2006 to 2024 were described as percentage increase or decrease in numbers of patients or procedures and, for comparisons of proportions, as percentage points (pp), i.e., arithmetic differences between the percentages. The correlation coefficient (*r*) was used to indicate the relationship between the annual number of TMD patients and all patients. Variation in TMD treatment volume was explained by treatment year and age group using linear regression modelling. Data handling, analyses and graphics were performed using Survo MM software (version 3.4.1; Survo Systems, Helsinki, Finland).

## 3 Results

The average annual number of TMD patients was 56 543, ranging from 42 148 in 2006 to 70 862 in 2015. Among all patients, the average annual number was 1 007 243, with yearly values ranging from 1 018 989 in 2006, peaking at 1 090 289 in 2013 and finally to 867 449 in 2024. The numbers of all patients and TMD patients were weakly correlated (*r* = 0.16). The numbers of all patients and TMD patients (both in thousands) are presented in Table 1. Over the 19 years observed, the annual proportion of TMD patients among all patients varied. The minimum value of 4.1% was in 2006, with peak values of 6.5%–6.7% in 2015–2019. From 2006 to 2024, the total number of patients decreased by 151 540 (14.9%), while the number of TMD patients increased by 9.7% and their proportion increased by 1.2 pp.

**TABLE 1:** Number of all patients and patients treated for temporomandibular disorders (TMD patients) in private oral healthcare in Finland by treatment year in 2006–2024.

Year	Patients	TMD patients	
	All × 1000	*n* × 1000	%
2006	1019	42.1	4.14
2007	1034	44.8	4.34
2008	1047	47.7	4.56
2009	1049	51.2	4.89
2010	1039	51.8	4.98
2011	1061	54.9	5.17
2012	1074	57.8	5.39
2013	1090	60.0	5.51
2014	1080	61.3	5.67
2015	1079	70.9	6.57
2016	1017	67.8	6.67
2017	998	66.6	6.68
2018	974	64.4	6.61
2019	966	62.8	6.50
2020	920	58.3	6.34
2021	936	58.3	6.23
2022	948	56.1	5.92
2023	940	51.2	5.44
2024	867	46.2	5.33
Mean	1007	56.5	5.61
Diff n	−152	+4.1	
Diff %	−14.87	+9.65	+1.19 pp

Abbreviations: Diff, difference from 2006 to 2024; pp, percentage points.

Proportions (%) of patients receiving TMD treatment by age group and treatment year are shown in Figure 1. All curves followed a similar shape, with differences by treatment year. The curves for 2017 and 2020 showed the highest values, while the 2024 curve was similar to the 2012 curve and partly below it. In all years, the 25–55‐year age groups had the highest proportions of TMD patients. The 30–50‐year age groups exceeded 7% in 2017 and 2020, and 6.5% in 2022, while in 2024 the same age groups were below 6%. No remarkable changes in TMD figures were found during the COVID‐19 pandemic years.

**FIGURE 1:**
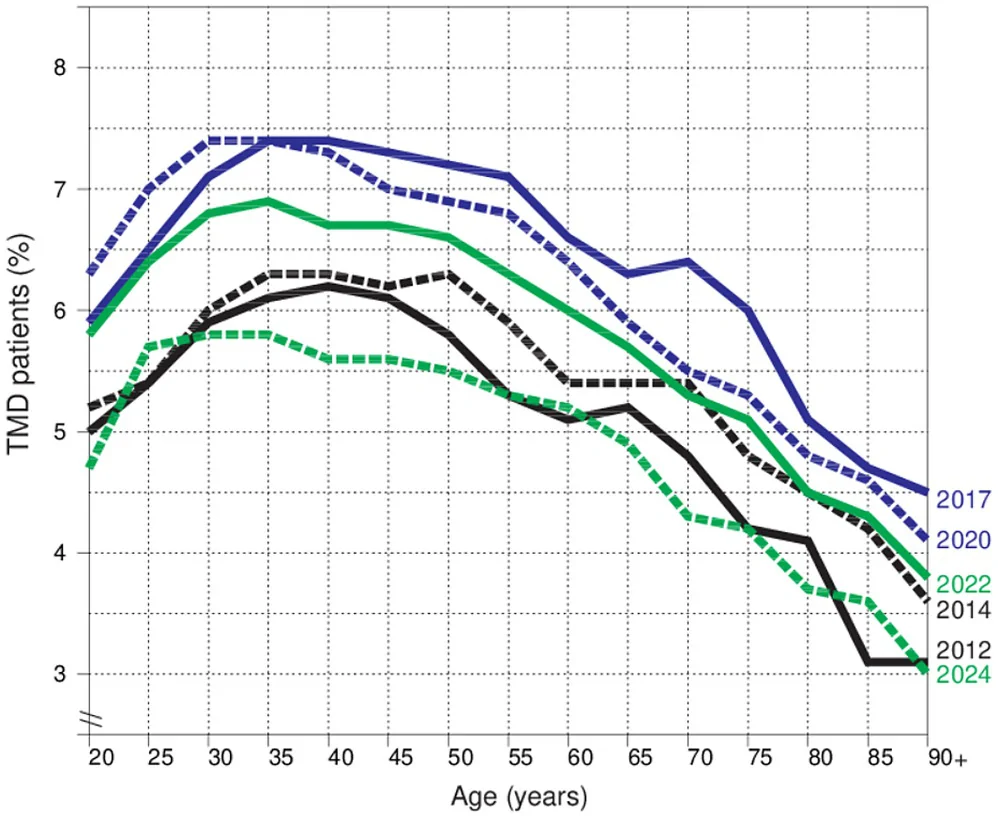
Patients (%) receiving treatment for temporomandibular disorders (TMD) among all private oral healthcare patients in Finland by age group and by treatment year.

The numbers of all clinical treatment procedures and those related to TMD are shown in Table 2. The number of TMD procedures (in thousands) was similar in 2006 and 2024 (55.5 and 55.7), with a peak of 90.5 in 2015. From 2006 to 2024, all clinical procedures decreased by 41.0%, while TMD procedures increased slightly by 0.4%. The proportion of TMD procedures among all clinical procedures increased from 1.3% in 2006 to 2.0% in 2024, peaking at 2.4% in 2020. The average increase over the 19 years was 0.82 pp. The mean number of TMD treatment procedures per TMD patient by year was 1.2. In 2012–2024, the corresponding weighted annual means for all and for each age group ranged from 1.2 to 1.3.

**TABLE 2:** Numbers and percentages of all clinical procedures and separately treatment procedures for temporomandibular disorders (TMD) in private oral healthcare in Finland in 2006–2024.

Year of treatment	Oral healthcare treatment procedures		
	Clinical, all	TMD only	TMD/Clinical
	*n* × 1000	*n* × 1000	%
2006	4735	55.5	1.17
2007	4804	58.1	1.21
2008	4861	61.8	1.27
2009	4840	66.7	1.38
2010	4405	67.0	1.52
2011	4445	70.5	1.59
2012	4464	74.5	1.67
2013	4444	77.0	1.73
2014	4352	78.3	1.80
2015	4216	90.5	2.15
2016	3638	85.8	2.36
2017	3591	84.4	2.35
2018	3384	81.1	2.40
2019	3297	78.9	2.39
2020	2983	72.8	2.44
2021	3035	73.8	2.43
2022	2995	69.8	2.33
2023	2887	62.8	2.18
2024	2795	55.7	1.99
Mean	3904	71.8	1.84
Diff n	−1940	+0.24	
Diff %	−40.97	+0.43	+0.82 pp

Abbreviations: Diff, difference from 2006 to 2024; pp, percentage points.

Table 3 shows numbers and distributions (%) of TMD procedures by type for selected years between 2012 and 2024. Among duration‐coded TMD procedures, short‐duration procedures accounted for 43.3% and medium for 28.8% across detailed years. The corresponding figures for long procedures or splint therapy were around 14%, with differences of 2.1–3.5 pp. The slight expansion in the appliance category reflects growth in splint therapy and the introduction of the increasing occlusal vertical dimension in 2021.

**TABLE 3:** Annual distributions (%) of the treatment procedures for temporomandibular disorders (TMD) by category, in private oral healthcare in Finland in 2012, 2017, 2020, 2022, and 2024.

TMD treatment categories	Treatment year					
	2012	2017	2020	2022	2024	Mean
Short (< 10 min)	38.0	46.0	43.7	44.0	44.9	43.3
Medium (10–20 min)	31.7	27.5	29.9	28.1	26.4	28.8
Long (20 + min)	16.8	14.3	12.5	12.2	13.8	14.0
Splint therapy	13.5	12.2	13.9	14.7	14.9	13.9
Total (%)	100.0	100.0	100.0	100.0	100.0	100.0
Procedures (*n*)	74 454	84 387	72 824	69 777	55 743	71 473

In 2024, 28%–32% of TMD patients aged 20–39 years and 25%–27% of those aged 40–54 years received splint therapy. In 2017, the corresponding figures were 19%–21% and 14%–19%, and in 2012, 27%–29% and 18%–24%, respectively. Figure 2 illustrates the distributions of the TMD treatment procedures by age group in 2012, 2017, and 2024. In these years, short‐duration TMD procedures were the most common and were more frequent in older age groups, whereas splint therapy was more common in the younger age groups. In 2024, splints accounted for 23%–26% of all TMD procedures among patients aged 20–39; in 2017 and 2012, the corresponding shares were 20%–22%.

**FIGURE 2:**
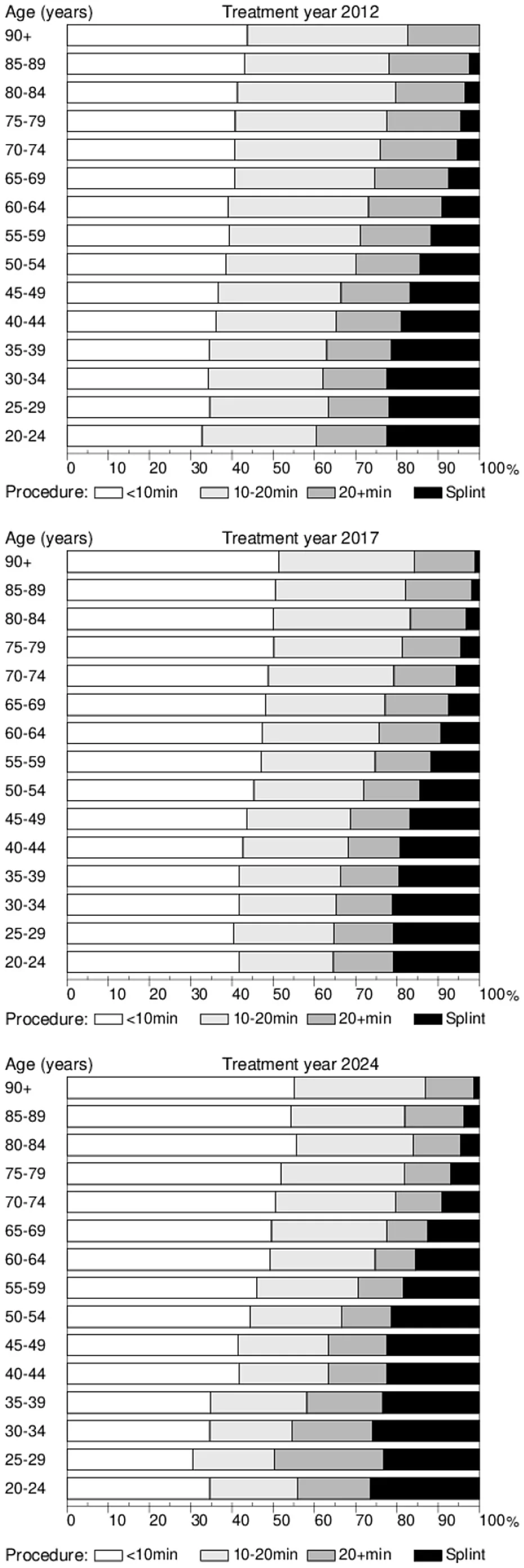
Distribution (%) of treatment procedures for temporomandibular disorders (TMD) by type and age group in private oral healthcare in Finland in 2012, 2017 and 2024.

Table 4 shows how patients' age and treatment year were associated with variation in TMD treatment volume. Linear regression models (A, B, C) demonstrated the strong effect of age group for each of the three volume descriptors analysed. Age group coefficients for (A) TMD patients as a percentage of all patients, (B) TMD procedures as a percentage of all procedures, and (C) splint applications as a percentage of all TMD procedures were all strong, negative and statistically significant (*p* < 0.001). Treatment year coefficients were smaller than those by age group but still statistically significant (*p* < 0.001) for the latter two descriptors (B, C).

**TABLE 4:** Variation in the number of patients and procedures for temporomandibular disorders (TMD) explained by linear regression of separate models (A, B, C) on data aggregated by age group, covering all adult patients treated in private oral healthcare in Finland in 2012–2024.

Dependent variables and explanation *R* ^2^	Explanatory factors	Regression coefficient (*β*)	Standard deviation	Beta	*t*	*p*
Model A						
TMD patients %	Age group	−0.036	0.004	−0.712	−10.25	< 0.001
*R* ^2^ 0.501	Time (year)	−0.006	0.020	−0.020	−0.282	n.s.
Model B						
TMD procedures %	Age group	−0.019	0.001	−0.783	−13.97	< 0.001
*R* ^2^ 0.679	Time (year)	0.036	0.008	0.256	4.568	< 0.001
Model C						
Splint therapy % of TMD	Age group	−0.358	0.009	−0.961	−39.35	< 0.001
*R* ^2^ 0.939	Time (year)	0.267	0.051	0.127	5.217	< 0.001

## 4 Discussion

### 4.1 Main Findings

The register‐based study analysed long‐term variation in TMD treatment volume among adults in subsidised private oral healthcare in Finland from 2006 to 2024. Throughout the 19‐year follow‐up period, TMD patients constituted a small but variable proportion of all patients. The findings did not support the null hypothesis. Peaks in the proportions of TMD patients and procedures were observed between 2015 and mid‐2022, with no noticeable changes during the COVID‐19 pandemic. Patient age played a significant role in treatment volume. The curves showed age‐related patterns across years and clear differences between years, with a gradual decline from 2017 to 2024 toward 2012 levels. In contrast, the number of patients receiving splint therapy increased steadily from 2017 onward, both in proportion and in the absolute number of appliances delivered.

In private oral healthcare, the number of TMD patients increased by 9.7% over the 19 years, while the total number of patients decreased by 14.9%. This increase may reflect greater awareness of TMD among dentists and patients, though definitive reasons cannot be determined without additional background information. The percentages of treated TMD patients provide only a rough proxy for prevalence of TMD, compared with recent global summaries [8, 9] and national studies from Finland [3, 4]. However, the present results closely resemble those of a recent three‐question Swedish survey [6].

The literature has long recommended caution in TMD treatment decisions. In 1992, Greene advised starting with simple, conservative and reversible treatments [15], and in 2010, continued to emphasize a conservative, patient‐centred approach prioritizing reversible and noninvasive treatments [16]. A comprehensive review suggests that treatment typically involves combinations of noninvasive therapies, patient education and counselling, behavioural therapy, medication, physical therapy and occlusal appliances [17]. However, a recent review concluded that very low‐certainty evidence did not show that splints reduce TMD pain [18], although noninvasive or conservative approaches appear to provide effective pain management and functional improvement [19]. A recent Cochrane review suggests splinting as a first‐line option when curative treatments are unavailable or when symptom relief is the primary goal, but found insufficient evidence to draw firm conclusions about the effectiveness of occlusal interventions for TMD [20].

This study showed that short‐duration procedures were the most frequently chosen option, comprising 38%–46% of the TMD procedures in the five years with detailed information. This prominence may indicate conservative approaches and caution in initiating more extensive TMD procedures. Unfortunately, the register codes do not specify content (counselling, exercises, medications), and the only specific TMD codes beyond duration are splint therapy or increasing occlusal vertical dimension. Generally, register‐based data include no description of the content of the procedure, the reason for treatment or the TMD diagnosis; such information, if recorded, resides only in the patient's health record. A Swedish dentist survey reported that many TMD treatments were provided without any pre‐treatment registrations [21], which may also be common practice in Finland.

Over the five years with detailed procedure information, the proportion of TMD patients receiving splint therapy remained fairly stable, averaging 13.9%, which exceeds figures from Västerbotten [11] and aligns with Stockholm [6]. Swedish dentist surveys reported a difference in the provision of splint therapy between the public and private sectors in 2010: 15% versus 36% [21] and in the public sector, the increase from 16% in 2010 to 23% in 2014 [22]. Interocclusal appliances were the treatment most dentists felt confident providing and reported having good clinical routines for [22]. In Finland, similar sectoral differences may exist. Cross‐sector comparisons were not possible here because the data cover only the private sector, while a previous Finnish report shows lower TMD procedure shares in the public sector [12]. This may indicate caution in TMD treatment decisions or varying attitudes toward splint therapy, especially given the debated therapeutic benefits of splints and the additional laboratory costs [23].

Variation in TMD treatment volume was strongly associated with patient age, whether measured as the proportion of TMD patients, the proportion of TMD procedures, or splint therapy use. It is also noteworthy that the COVID‐19 pandemic did not produce significant changes in annual TMD treatment volumes. The linear regression results and graphic trends support prior reports from Sweden [5, 6]. However, age differences in TMD prevalence or treatment have rarely been reported; one review noted that estimated treatment needs were higher in younger adults (19–45 years) than in older adults [10].

### 4.2 Strengths and Limitations

The strengths of this study include its nearly two‐decade longitudinal scope, which enabled identification of both absolute and relative changes in TMD treatment patterns, and its comprehensive population‐wide registry data, which minimise bias from missing cases and systematic data collection errors. Thus, the study provides reliable real‐world information on TMD treatment volume and age distribution.

Limitations stem from the duration‐based TMD procedure codes, with a lack of detailed content for most procedures. Consequently, the study could not analyse patient‐reported factors, clinical diagnoses, or reasons underlying treatment decisions.

### 4.3 Conclusions

This longitudinal registry study of private oral healthcare in Finland shows that the proportion of TMD treatment increased modestly from 2006 to 2024, even though the total number of patients decreased. Treatment was consistently most common in younger adults; the pandemic had little impact on volumes and short‐duration, conservative measures and splint therapy played substantial roles. An increase in splint therapy may reflect increased dentist confidence and patient acceptance for splints. Registry content limitations preclude conclusions about diagnoses, indications and treatment effectiveness. Future studies using patient‐level data are needed, particularly to assess the basis for treatment decisions and the clinical effectiveness of TMD therapy.

## Author Contributions

**Maria Valaste:** methodology, writing – review and editing, data curation. **Kaija Hiltunen:** conceptualization, validation, visualization, writing – review and editing, writing – original draft. **Miira M. Vehkalahti:** conceptualization, investigation, writing – original draft, methodology, validation, visualization, writing – review and editing, formal analysis, data curation.

## Funding

Open access funded by Helsinki University Library.

## Conflicts of Interest

The authors declare no conflicts of interest.

## Data Availability

The data analysed in this study are not publicly available but are available from the corresponding author upon reasonable request.
